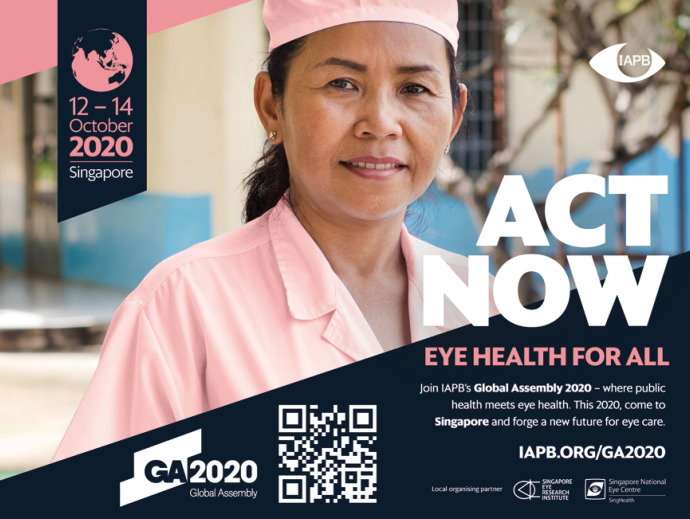# Ebola and the eye

**Published:** 2020-03-30

**Authors:** Gerry Clare

**Affiliations:** 1 Ophthalmologist with Médecins sans Frontières in Liberia during the 2014 Ebola outbreak.

**Survivors of Ebola virus infection may present late with a spectrum of ocular manifestations, ranging from mild retinal scarring to complete bilateral blindness resulting from tractional retinal detachment, cataract and glaucoma secondary to intraocular inflammation.**

An infectious disease specialist's brush with death during the 2014 Ebola crisis in West Africa proved instructive to ophthalmologists.^1^ On becoming severely ill with signs of having contracted Ebola virus disease (EVD), the doctor was evacuated to an isolation facility in the United States for supportive care, where he eventually recovered. A few weeks later, he noticed blurring of vision, pain and redness in one eye, and was diagnosed with uveitis. An aqueous sample from the affected eye was sent for analysis, and polymerase chain reaction (PCR) assay demonstrated the presence of replicating Ebola virus.

## *Ebolavirus* and other viruses causing uveitis

Of the six species in the *Ebolavirus* (EBOV) genus, four infect humans, causing an acute haemorrhagic fever that is associated with very high case fatality. The finding of replicating Ebola virus in aqueous is not surprising: Marburg virus, a member of the same *Filoviridae* family (enveloped, filamentous virions with a negative sense RNA genome), had been isolated from the intraocular fluid of infected persons several years before.^2^ Moreover, in previous outbreaks, Ebola virus infection had been noted to precipitate uveitis in a minority of survivors.^3^ In addition, *Flaviviridae* such as Zika, Dengue and West Nile viruses, and *Togaviridae* such as Chikungunya also cause intraocular inflammation.^4^

**“Ocular complications form part of post-Ebola virus disease syndrome, which also includes symptoms of joint and muscle pain and sometimes neurological problems.”**

## Ocular complications of Ebola virus disease

Ocular complications form part of post-Ebola virus disease syndrome, which also includes symptoms of joint and muscle pain and sometimes neurological problems.

A longitudinal study in Liberia established that EVD survivors have a significantly higher incidence of uveitis than a control group of close contacts.^4^ Uveitis is the most common ocular complication of EVD, affecting nearly one third of people recovering from EVD.

**Figure 1:**
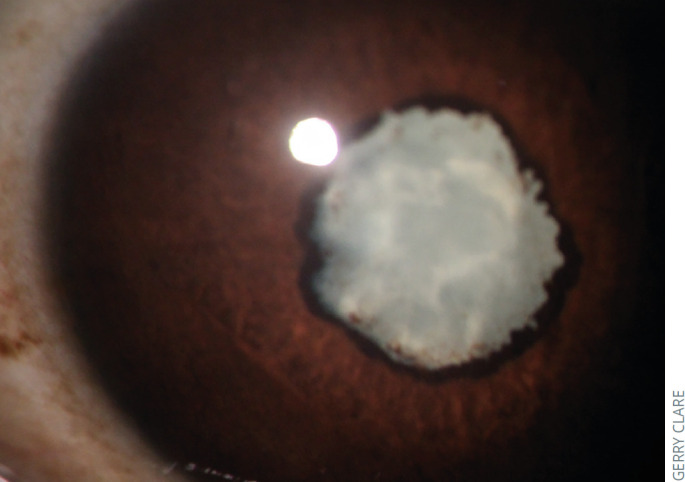
A dense white cataract masking a tractional retinal detachment in a young Liberian female Ebola survivor. Note the irregular pupil margin, indicating posterior synechiae. She was blind in both eyes.

A range of signs are possible, including non-granulomatous anterior uveitis, posterior synechiae, iris atrophy, vitreous inflammation, chorioretinal scarring, panuveitis and optic neuropathy. These findings reflect direct viral invasion of the normally immune-privileged intraocular space. Inflammation can range from mild to severe, causing raised intraocular pressure, cataract (Figure 1), tractional retinal detachment and phthisis, and may affect both eyes, causing visual impairment or complete blindness.^5^

**Figure 2:**
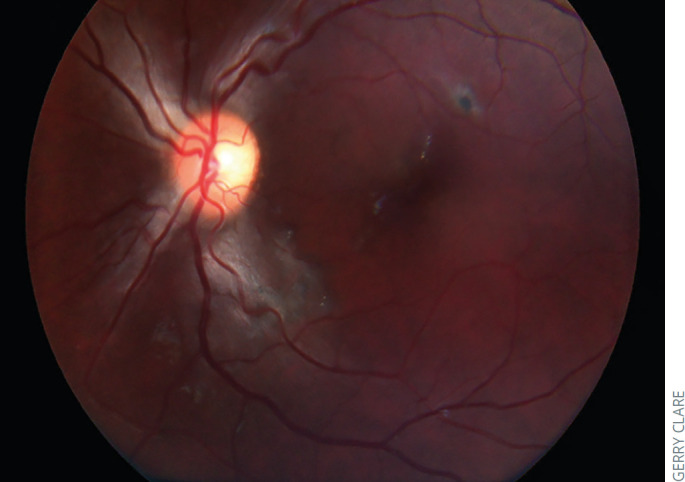
A small pigmented scar with a hypopigmented halo is all that remains to indicate past ocular inflammation in this young Ebola survivor.

A variety of chorioretinal lesions may be found in survivors, such as pigmented retinal scars with a hypopigmented halo (Figure 2).^6^ In a study of 50 survivors, no evidence of viral persistence in the eye was found at a median of 19 months after diagnosis, indicating that cataract surgery could be undertaken safely.^7^,^8^

## Management of uveitis resulting from Ebola

Treatment options include topical or systemic ocular antihypertensives, cycloplegic and mydriatic agents for analgesia and to prevent synechiae, and topical corticosteroids.

The role of antiviral drugs in eliminating intraocular infection with Ebola virus is currently being evaluated.

## Implications

In those recovering from Ebola infection, the finding of treatable eye disease which can lead to visual impairment has two implications for organisations providing health care to Ebola survivors.

### 1. Offer follow-up eye examinations to all Ebola survivors

Health care workers should be prepared to assess the eye, which should include:

- Assessing vision
- Examining the fundus
- Measuring intraocular pressure.

Health workers can use a smartphone app to measure visual acuity, with the addition of a camera adaptor clip to visualise the fundus.^9^ With training, intraocular pressure can be measured using portable instruments.

### 2. Develop treatment and referral pathways

Adequate treatment and referral pathways should be developed, potentially using retinal images for remote consultation with an ophthalmologist.

Happily, the infectious disease specialist who developed Ebola uveitis partially recovered his vision, but others are not so lucky.

Recent Ebola outbreaks have had the unexpected effect of exposing the striking shortage of ophthalmic services available in affected communities, as well as the lack of preparedness to deal with its ophthalmic consequences. For instance, some clinicians have been reluctant to treat survivors with cataract due to unfounded fears over persisting infection. This adds to the already overwhelming burden on health services in countries struggling with the aftermath of an Ebola epidemic.

**Figure:**